# Crystal structure of poly[[hexa­qua-1κ^4^ *O*,2κ^2^ *O*-bis­(μ_3_-pyridine-2,4-di­car­box­ylato-1κ*O* ^2^:2κ^2^ *N*,*O* ^2′^;1′κ*O* ^4^)cobalt(II)­strontium(II)] dihydrate]

**DOI:** 10.1107/S2056989015014942

**Published:** 2015-08-15

**Authors:** Zhaojun Yu, Peng Jiang, Yanmei Chen

**Affiliations:** a College of Chemical Engineering, Huanggang Normal University, Huanggang 438000, People’s Republic of China

**Keywords:** crystal structure, heterometallic complex, pyridine-2,4-di­carb­oxy­lic acid heterometallic complex

## Abstract

In the title polymeric complex, {[CoSr(C_7_H_3_NO_4_)_2_(H_2_O)_6_]·2H_2_O}_*n*_, the Co^II^ ion, which is situated on a crystallographic centre of inversion, is six-coordinated by two O atoms and two N atoms from two pyridine-2,4-di­carboxyl­ate (pydc^2−^) ligands and two terminal water mol­ecules in a slightly distorted octa­hedral geometry, to form a *trans*-[Co(pydc)_2_(H_2_O)_2_]^2−^ unit. The Sr^II^ ion, situated on a *C*_2_ axis, is coordinated by four O atoms from four pydc^2−^ ligands and four water mol­ecules. The coordination geometry of the Sr^II^ atom can be best described as a distorted dodeca­hedron. Each Sr^II^ ion bridges four [Co(pydc)_2_(H_2_O)_2_]^2−^ units by four COO^−^ groups of four pydc^2−^ ligands to form a three-dimensional network structure. Two additional solvent water mol­ecules are observed in the crystal structure and are connected to the three-dimensional coordination polymer by O—H⋯O hydrogen bonds. Further intra- and intermolecular O—H⋯O hydrogen bonds consolidate the overall structure.

## Related literature   

For similar heterometallic complexes, see: Chen *et al.* (2014 ▸, 2015 ▸); Gil de Muro *et al.* (1999 ▸); Li *et al.* (1989 ▸); Mege-Revil & Price (2013 ▸); Zasurskaya *et al.* (2000 ▸, 2001 ▸, 2006 ▸); Zhang (1993 ▸); Zhang *et al.* (1992 ▸).
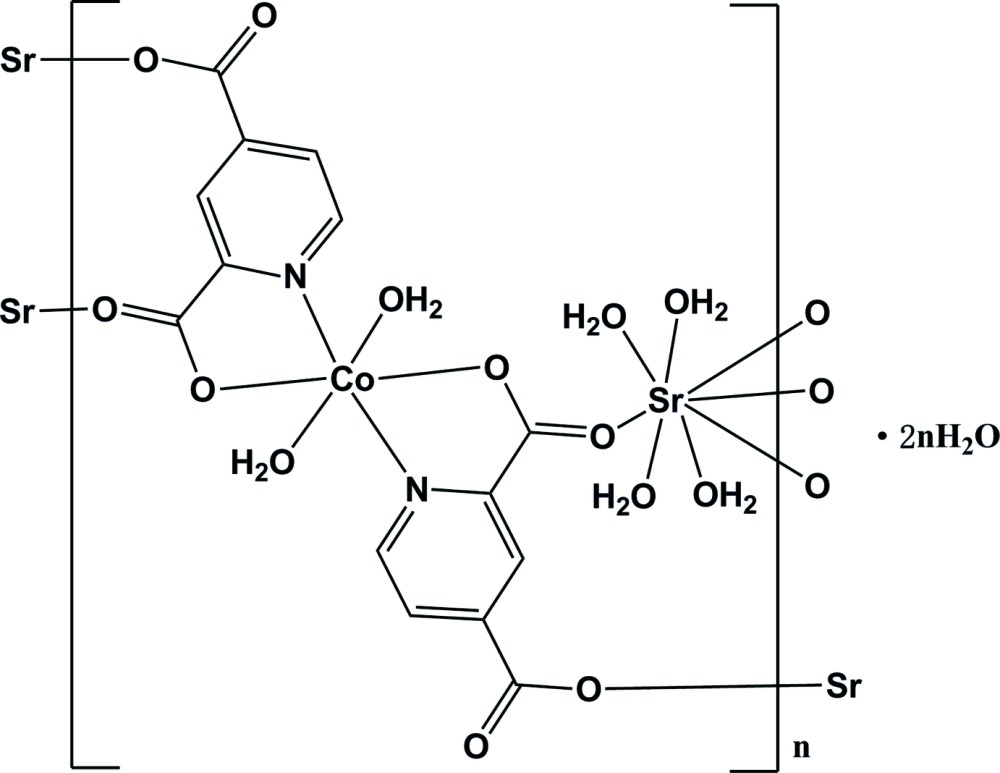


## Experimental   

### Crystal data  

[CoSr(C_7_H_3_NO_4_)_2_(H_2_O)_6_]·2H_2_O
*M* *_r_* = 620.89
Monoclinic, 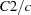

*a* = 18.628 (4) Å
*b* = 6.8742 (14) Å
*c* = 19.101 (4) Å
β = 118.77 (3)°
*V* = 2144.0 (8) Å^3^
*Z* = 4
Mo *K*α radiation
μ = 3.35 mm^−1^
*T* = 293 K
0.20 × 0.18 × 0.15 mm

### Data collection  

Bruker APEXII CCD diffractometer
Absorption correction: multi-scan (*SADABS*; Bruker, 2005 ▸) *T*_min_ = 0.554, *T*_max_ = 0.634
14907 measured reflections
1892 independent reflections
1776 reflections with *I* > 2σ(*I*)
*R*_int_ = 0.023

### Refinement  

*R*[*F* ^2^ > 2σ(*F* ^2^)] = 0.020
*wR*(*F* ^2^) = 0.055
*S* = 1.04
1892 reflections
180 parameters
H atoms treated by a mixture of independent and constrained refinement
Δρ_max_ = 0.25 e Å^−3^
Δρ_min_ = −0.46 e Å^−3^

### 

Data collection: *APEX2* (Bruker, 2005 ▸); cell refinement: *SAINT* (Bruker, 2005 ▸); data reduction: *SAINT*; program(s) used to solve structure: *SHELXS97* (Sheldrick, 2008 ▸); program(s) used to refine structure: *SHELXL97* (Sheldrick, 2008 ▸); molecular graphics: *SHELXTL* (Sheldrick, 2008 ▸) and *DIAMOND* (Brandenburg, 1999 ▸); software used to prepare material for publication: *SHELXTL*.

## Supplementary Material

Crystal structure: contains datablock(s) I. DOI: 10.1107/S2056989015014942/im2469sup1.cif

Structure factors: contains datablock(s) I. DOI: 10.1107/S2056989015014942/im2469Isup2.hkl

**Fig. 1.**
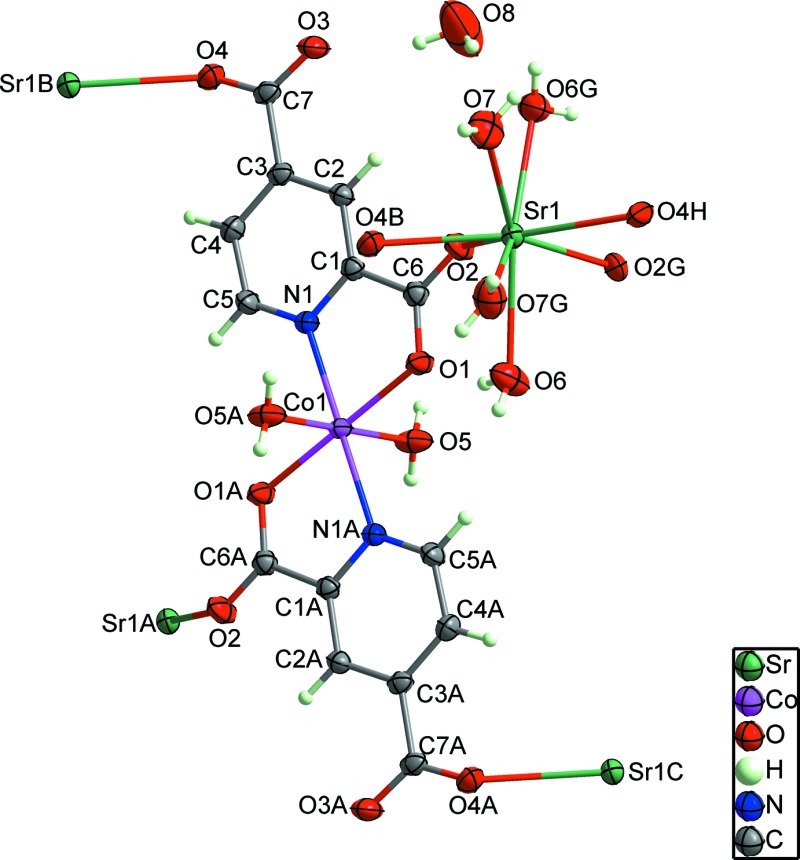
The molecular structure of the title compound. Displacement ellipsoids are drawn at the 50% probability level. Symmetry codes: A: -x,-y + 1,-z + 1; B: -x + 1/2,-y + 1/2,-z + 1; C: x - 1/2,y + 1/2,z; G: -x,y,-z + 1/2; H: x - 1/2,1/2 - y,z - 1/2.

**Fig. 2.**
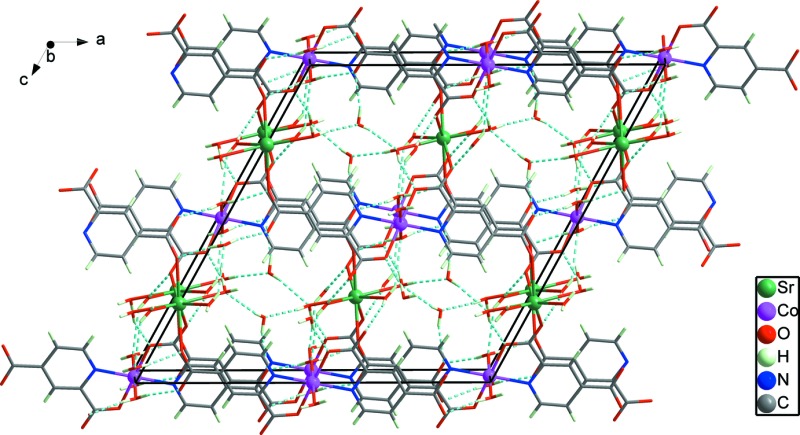
The packing diagram for the title compound, viewed down the b-axis, with hydrogen bonds drawn as dashed lines.

Click here for additional data file.x y z x y z x y z x y z x y z . DOI: 10.1107/S2056989015014942/im2469fig1.tif
The mol­ecular structure of the title compound. Displacement ellipsoids are drawn at the 50% probability level. Symmetry codes: A: −*x*,-*y* + 1,-*z* + 1; B: −*x* + 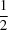,-*y* + 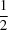,-*z* + 1; C: *x* − 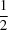,*y* + 1/2,*z*; G: −*x*,*y*,-*z* + 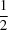; H: *x* − 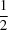,1/2 − *y*,*z* − 1/2.

b . DOI: 10.1107/S2056989015014942/im2469fig2.tif
The packing diagram for the title compound, viewed down the *b*-axis, with hydrogen bonds drawn as dashed lines.

CCDC reference: 761895

Additional supporting information:  crystallographic information; 3D view; checkCIF report

## Figures and Tables

**Table 1:** Hydrogen-bond geometry (, )

*D*H*A*	*D*H	H*A*	*D* *A*	*D*H*A*
O5H5*A*O4^i^	0.78(3)	2.00(3)	2.7672(19)	168(2)
O5H5*B*O3^ii^	0.79(3)	1.96(3)	2.734(2)	165(3)
O6H6*B*O8^iii^	0.77(3)	2.07(4)	2.833(3)	173(3)
O7H7*A*O5^iv^	0.77(3)	2.57(3)	3.233(3)	145(3)
O7H7*B*O2^v^	0.85(4)	2.39(3)	3.170(2)	153(3)
O8H8*A*O6^vi^	0.75(5)	2.50(5)	3.238(3)	167(5)
O8H8*B*O3	0.82(6)	2.04(6)	2.831(3)	164(6)
O6H6*A*O1	0.77(3)	2.03(3)	2.781(2)	164(3)

Symmetry codes: (i) 
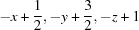
; (ii) 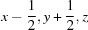; (iii) 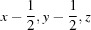; (iv) 
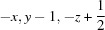
; (v) 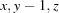; (vi) 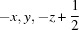.

## supplementary crystallographic information

### S1. Introduction

As an N,O-containing ligand, pyridine-2,4-di­carb­oxy­lic acid (pydc) possesses different binding sites with specific affinities to discriminate different metal ions. It coordinates to alkaline earth metals by using the oxygen atoms to form 'complex-ligands', and the 'complex-ligands' then bind with other metal ions to generate heterometallic coordination polymers. In our previous work, two heterometallic complexes containing Co^II^ and Sr^II^ ions were reported (Chen *et al.*, 2015; Chen *et al.*, 2014). As a continuation of our previous work, in this paper a new Co—Sr-pydc complex is reported.

### S2.1. Synthesis and crystallization

A mixture of pyridine-2,4-di­carb­oxy­lic acid (0.0337 g, 0.2 mmol), Sr(OH)_2_·8 H_2_O (0.0262 g, 0.1 mmol), Co(OAc)_2_·4H_2_O (0.0244 g,0.1 mmol), and H_2_O (2 ml) was sealed in a pyrex-bottle (8 ml) and heated to 90°C for 2 days. The tube was then cooled to room temperature, generating yellow rod crystals. Yield: 0.0225 g (37%, based on Co). Elemental analysis calc. for C_14_H_22_CoN_2_O_16_Sr: C, 27.08; H, 3.57; N, 4.51%. Found: C,27.32; H, 3.57; N, 4.53%.

### S2.2. Refinement

H atoms bonded to C atoms of the pyridine ring were positioned geometrically and refined as riding atoms, with C—H = 0.97 Å, and with U_iso_(H) = 1.2 U_eq_(C). The water H atoms were located from a difference Fourier map and refined with restraints of O—H = 0.86 (1) Å and U_iso_(H) = 1.5 U_eq_(O).

### S3. Results and discussion

In the title polymeric complex, [C_14_H_18_CoN_2_O_14_Sr]n·2n H_2_O, the Co^II^ ion, which is situated on a crystallographic centre of inversion, is six-coordinated by two oxygen atoms and two nitro­gen atoms from two pyridine-2,4-di­carboxyl­ate (pydc^2-^) ligands and two terminal water molecules in a slightly distorted o­cta­hedral geometry, to form a *trans*-[Co(pydc)_2_(H_2_O)_2_]^2-^ unit. The Sr^II^ ion being situated on the *C*_2_-axis is coordinated by four oxygen atoms from four pydc^2-^ ligands and four water molecules. The coordination geometry of the Sr^II^ atom can be best described as a distorted dodecahedron. Each Sr^II^ ion bridges four [Co(pydc)_2_(H_2_O)_2_]^2-^ units by four COO^-^ groups of four pydc^2-^ ligands to form a three-dimensional network structure. The structure is also built up by hydrogen bond inter­actions between coordinated water molecules and carb­oxy­lic groups of pydc^2-^ ligands. Sovent water molecules are connected to the 3-D network by three inter­molecular hydrogen bond inter­actions O6—H6B···O8, O8—H8A···O6 and O3—H8B···O3.

### Crystal data

**Table:**

[CoSr(C_7_H_3_NO_4_)_2_(H_2_O)_6_]·2H_2_O	*F*(000) = 1252
*M**_r_* = 620.89	*D*_x_ = 1.924 Mg m^−^^3^
Monoclinic, *C*2/*c*	Mo *K*α radiation, λ = 0.71073 Å
Hall symbol: -C 2yc	Cell parameters from 1892 reflections
*a* = 18.628 (4) Å	θ = 2.4–25.0°
*b* = 6.8742 (14) Å	µ = 3.35 mm^−^^1^
*c* = 19.101 (4) Å	*T* = 293 K
β = 118.77 (3)°	Block, yellow
*V* = 2144.0 (8) Å^3^	0.20 × 0.18 × 0.15 mm
*Z* = 4	

### Data collection

**Table:**

Bruker APEXII CCD diffractometer	1892 independent reflections
Radiation source: fine-focus sealed tube	1776 reflections with *I* > 2σ(*I*)
Graphite monochromator	*R*_int_ = 0.023
φ and ω scans	θ_max_ = 25.0°, θ_min_ = 2.4°
Absorption correction: multi-scan (*SADABS*; Bruker, 2005)	*h* = −22→22
*T*_min_ = 0.554, *T*_max_ = 0.634	*k* = −7→8
14907 measured reflections	*l* = −22→22

### Refinement

**Table:**

Refinement on *F*^2^	Primary atom site location: structure-invariant direct methods
Least-squares matrix: full	Secondary atom site location: difference Fourier map
*R*[*F*^2^ > 2σ(*F*^2^)] = 0.020	Hydrogen site location: inferred from neighbouring sites
*wR*(*F*^2^) = 0.055	H atoms treated by a mixture of independent and constrained refinement
*S* = 1.04	*w* = 1/[σ^2^(*F*_o_^2^) + (0.032*P*)^2^ + 2.0752*P*] where *P* = (*F*_o_^2^ + 2*F*_c_^2^)/3
1892 reflections	(Δ/σ)_max_ < 0.001
180 parameters	Δρ_max_ = 0.25 e Å^−^^3^
0 restraints	Δρ_min_ = −0.46 e Å^−^^3^

### Special details

**Table:**

Geometry. All e.s.d.'s (except the e.s.d. in the dihedral angle between two l.s. planes) are estimated using the full covariance matrix. The cell e.s.d.'s are taken into account individually in the estimation of e.s.d.'s in distances, angles and torsion angles; correlations between e.s.d.'s in cell parameters are only used when they are defined by crystal symmetry. An approximate (isotropic) treatment of cell e.s.d.'s is used for estimating e.s.d.'s involving l.s. planes.
Refinement. Refinement of *F*^2^ against ALL reflections. The weighted *R*-factor *wR* and goodness of fit *S* are based on *F*^2^, conventional *R*-factors *R* are based on *F*, with *F* set to zero for negative *F*^2^. The threshold expression of *F*^2^ > σ(*F*^2^) is used only for calculating *R*-factors(gt) *etc*. and is not relevant to the choice of reflections for refinement. *R*-factors based on *F*^2^ are statistically about twice as large as those based on *F*, and *R*- factors based on ALL data will be even larger. During the refinement, the command 'omit -3 50' was used to omit the reflections above 50 degree.

### Fractional atomic coordinates and isotropic or equivalent isotropic displacement parameters (Å^2^)

**Table:**

	*x*	*y*	*z*	*U*_iso_*/*U*_eq_	
Sr1	0.0000	0.01264 (3)	0.2500	0.02146 (10)	
Co1	0.0000	0.5000	0.5000	0.01990 (11)	
O1	−0.00844 (7)	0.38635 (19)	0.39640 (7)	0.0266 (3)	
O2	0.06342 (8)	0.3272 (2)	0.33384 (7)	0.0315 (3)	
O4	0.42018 (7)	0.5817 (2)	0.60160 (7)	0.0305 (3)	
O3	0.36287 (8)	0.4710 (2)	0.47716 (9)	0.0317 (3)	
O5	−0.03083 (9)	0.7721 (2)	0.44277 (9)	0.0331 (3)	
H5A	0.0016 (16)	0.826 (4)	0.4351 (14)	0.050*	
H5B	−0.0549 (15)	0.844 (4)	0.4563 (14)	0.050*	
O6	−0.11541 (10)	0.1289 (3)	0.28103 (10)	0.0449 (4)	
H6A	−0.0932 (18)	0.204 (5)	0.3145 (18)	0.067*	
H6B	−0.143 (2)	0.067 (5)	0.2920 (19)	0.067*	
O7	0.07800 (11)	−0.3050 (3)	0.24138 (10)	0.0465 (4)	
H7A	0.0880 (19)	−0.302 (5)	0.2065 (18)	0.070*	
H7B	0.0788 (19)	−0.423 (5)	0.2552 (18)	0.070*	
N1	0.12275 (9)	0.5227 (2)	0.52456 (9)	0.0200 (3)	
C1	0.13299 (11)	0.4627 (2)	0.46297 (10)	0.0186 (3)	
C2	0.20789 (11)	0.4628 (2)	0.46526 (10)	0.0203 (4)	
H2	0.2125	0.4217	0.4212	0.024*	
C3	0.27660 (11)	0.5250 (2)	0.53410 (11)	0.0199 (4)	
C4	0.26621 (10)	0.5851 (3)	0.59769 (10)	0.0243 (4)	
H4	0.3108	0.6272	0.6448	0.029*	
C5	0.18875 (10)	0.5820 (3)	0.59040 (10)	0.0250 (4)	
H5	0.1824	0.6234	0.6335	0.030*	
C6	0.05723 (10)	0.3868 (2)	0.39153 (10)	0.0210 (4)	
C7	0.35996 (11)	0.5254 (2)	0.53790 (11)	0.0222 (4)	
O8	0.27133 (18)	0.4156 (6)	0.30992 (15)	0.1108 (11)	
H8A	0.239 (3)	0.339 (8)	0.295 (3)	0.166*	
H8B	0.289 (3)	0.430 (8)	0.358 (3)	0.166*	

### Atomic displacement parameters (Å^2^)

**Table:**

	*U*^11^	*U*^22^	*U*^33^	*U*^12^	*U*^13^	*U*^23^
Sr1	0.02048 (15)	0.02650 (15)	0.01701 (14)	0.000	0.00871 (11)	0.000
Co1	0.01467 (19)	0.0272 (2)	0.01995 (19)	−0.00072 (11)	0.01003 (15)	−0.00234 (12)
O1	0.0171 (6)	0.0390 (7)	0.0237 (6)	−0.0033 (5)	0.0099 (5)	−0.0083 (5)
O2	0.0295 (7)	0.0452 (8)	0.0233 (6)	−0.0070 (6)	0.0154 (6)	−0.0120 (6)
O4	0.0171 (6)	0.0449 (8)	0.0262 (7)	−0.0022 (6)	0.0079 (5)	0.0046 (6)
O3	0.0247 (7)	0.0431 (8)	0.0344 (8)	0.0008 (6)	0.0199 (6)	−0.0043 (6)
O5	0.0305 (7)	0.0300 (8)	0.0512 (9)	0.0033 (6)	0.0295 (7)	0.0058 (6)
O6	0.0445 (9)	0.0566 (11)	0.0438 (9)	−0.0198 (7)	0.0294 (8)	−0.0190 (8)
O7	0.0529 (10)	0.0425 (9)	0.0410 (9)	0.0015 (8)	0.0201 (8)	−0.0091 (7)
N1	0.0175 (8)	0.0242 (8)	0.0202 (8)	0.0004 (5)	0.0106 (6)	−0.0009 (5)
C1	0.0190 (8)	0.0183 (8)	0.0198 (9)	0.0007 (7)	0.0103 (7)	0.0015 (7)
C2	0.0218 (9)	0.0213 (8)	0.0217 (9)	0.0014 (7)	0.0135 (7)	0.0002 (7)
C3	0.0181 (9)	0.0183 (8)	0.0247 (9)	0.0024 (6)	0.0115 (8)	0.0048 (6)
C4	0.0191 (8)	0.0303 (10)	0.0208 (8)	−0.0007 (7)	0.0075 (7)	−0.0007 (7)
C5	0.0216 (9)	0.0353 (10)	0.0198 (8)	0.0000 (8)	0.0114 (7)	−0.0052 (7)
C6	0.0213 (8)	0.0206 (9)	0.0208 (8)	0.0007 (7)	0.0099 (7)	0.0004 (6)
C7	0.0184 (9)	0.0214 (9)	0.0278 (10)	0.0032 (7)	0.0119 (8)	0.0072 (7)
O8	0.0901 (19)	0.191 (3)	0.0507 (13)	−0.065 (2)	0.0334 (14)	−0.0089 (17)

### Geometric parameters (Å, º)

**Table:**

Co1—O1^i^	2.0619 (12)	O7—H7B	0.85 (4)
Co1—O1	2.0619 (12)	N1—C5	1.331 (2)
Co1—O5	2.1025 (15)	N1—C1	1.344 (2)
Co1—O5^i^	2.1025 (15)	C1—C2	1.375 (3)
Co1—N1	2.1072 (16)	C1—C6	1.507 (2)
Co1—N1^i^	2.1072 (16)	C2—C3	1.389 (3)
O1—C6	1.271 (2)	C2—H2	0.9300
O2—C6	1.232 (2)	C3—C4	1.382 (3)
O4—C7	1.255 (2)	C3—C7	1.519 (2)
O3—C7	1.245 (2)	C4—C5	1.382 (2)
O5—H5A	0.78 (3)	C4—H4	0.9300
O5—H5B	0.79 (3)	C5—H5	0.9300
O6—H6A	0.77 (3)	O8—H8A	0.75 (5)
O6—H6B	0.77 (3)	O8—H8B	0.82 (6)
O7—H7A	0.77 (3)		
			
O1^i^—Co1—O1	180.0	C1—N1—Co1	112.24 (12)
O1^i^—Co1—O5	92.14 (6)	N1—C1—C2	122.78 (16)
O1—Co1—O5	87.86 (6)	N1—C1—C6	115.66 (15)
O1^i^—Co1—O5^i^	87.86 (6)	C2—C1—C6	121.51 (15)
O1—Co1—O5^i^	92.14 (6)	C1—C2—C3	119.27 (16)
O5—Co1—O5^i^	180.0	C1—C2—H2	120.4
O1^i^—Co1—N1	100.66 (6)	C3—C2—H2	120.4
O1—Co1—N1	79.34 (6)	C4—C3—C2	117.96 (16)
O5—Co1—N1	92.61 (6)	C4—C3—C7	121.99 (16)
O5^i^—Co1—N1	87.39 (6)	C2—C3—C7	120.05 (16)
O1^i^—Co1—N1^i^	79.34 (6)	C5—C4—C3	119.22 (16)
O1—Co1—N1^i^	100.66 (6)	C5—C4—H4	120.4
O5—Co1—N1^i^	87.39 (6)	C3—C4—H4	120.4
O5^i^—Co1—N1^i^	92.61 (6)	N1—C5—C4	123.02 (16)
N1—Co1—N1^i^	180.0	N1—C5—H5	118.5
C6—O1—Co1	115.97 (11)	C4—C5—H5	118.5
Co1—O5—H5A	118.5 (19)	O2—C6—O1	124.93 (16)
Co1—O5—H5B	116.3 (18)	O2—C6—C1	118.35 (15)
H5A—O5—H5B	112 (3)	O1—C6—C1	116.71 (14)
H6A—O6—H6B	108 (3)	O3—C7—O4	125.23 (17)
H7A—O7—H7B	109 (3)	O3—C7—C3	117.19 (17)
C5—N1—C1	117.74 (15)	O4—C7—C3	117.58 (16)
C5—N1—Co1	130.01 (12)	H8A—O8—H8B	109 (5)
			
O5—Co1—O1—C6	90.49 (13)	C7—C3—C4—C5	179.75 (16)
N1—Co1—O1—C6	−2.57 (12)	C1—N1—C5—C4	0.3 (3)
O5—Co1—N1—C5	96.46 (16)	Co1—N1—C5—C4	178.77 (13)
O1—Co1—N1—C1	2.35 (11)	C3—C4—C5—N1	0.3 (3)
O5—Co1—N1—C1	−84.97 (12)	Co1—O1—C6—O2	−179.05 (14)
C5—N1—C1—C2	−0.8 (3)	Co1—O1—C6—C1	2.30 (19)
Co1—N1—C1—C2	−179.57 (13)	N1—C1—C6—O2	−178.92 (16)
C5—N1—C1—C6	176.84 (15)	C2—C1—C6—O2	−1.3 (3)
Co1—N1—C1—C6	−1.93 (18)	N1—C1—C6—O1	−0.2 (2)
N1—C1—C2—C3	0.8 (3)	C2—C1—C6—O1	177.49 (16)
C6—C1—C2—C3	−176.73 (15)	C4—C3—C7—O3	−179.09 (16)
C1—C2—C3—C4	−0.2 (2)	C2—C3—C7—O3	1.0 (2)
C1—C2—C3—C7	179.75 (15)	C4—C3—C7—O4	0.4 (2)
C2—C3—C4—C5	−0.3 (3)	C2—C3—C7—O4	−179.53 (16)

Symmetry code: (i) −*x*, −*y*+1, −*z*+1.

### Hydrogen-bond geometry (Å, º)

**Table:**

*D*—H···*A*	*D*—H	H···*A*	*D*···*A*	*D*—H···*A*
O5—H5*A*···O4^ii^	0.78 (3)	2.00 (3)	2.7672 (19)	168 (2)
O5—H5*B*···O3^iii^	0.79 (3)	1.96 (3)	2.734 (2)	165 (3)
O6—H6*B*···O8^iv^	0.77 (3)	2.07 (4)	2.833 (3)	173 (3)
O7—H7*A*···O5^v^	0.77 (3)	2.57 (3)	3.233 (3)	145 (3)
O7—H7*B*···O2^vi^	0.85 (4)	2.39 (3)	3.170 (2)	153 (3)
O8—H8*A*···O6^vii^	0.75 (5)	2.50 (5)	3.238 (3)	167 (5)
O8—H8*B*···O3	0.82 (6)	2.04 (6)	2.831 (3)	164 (6)
O6—H6*A*···O1	0.77 (3)	2.03 (3)	2.781 (2)	164 (3)

Symmetry codes: (ii) −*x*+1/2, −*y*+3/2, −*z*+1; (iii) *x*−1/2, *y*+1/2, *z*; (iv) *x*−1/2, *y*−1/2, *z*; (v) −*x*, *y*−1, −*z*+1/2; (vi) *x*, *y*−1, *z*; (vii) −*x*, *y*, −*z*+1/2.
