# Supplementary figures and images for: Crystal structure of poly[[hexa­qua-1κ4 O,2κ2 O-bis­(μ3-pyridine-2,4-di­car­box­ylato-1κO 2:2κ2 N,O 2′;1′κO 4)cobalt(II)­strontium(II)] dihydrate]
Source: Acta Crystallogr E Crystallogr Commun. 2015 Aug 15;71(Pt 9):m167–8. doi: 10.1107/S2056989015014942 (PMC4555429; doi:10.1107/S2056989015014942)

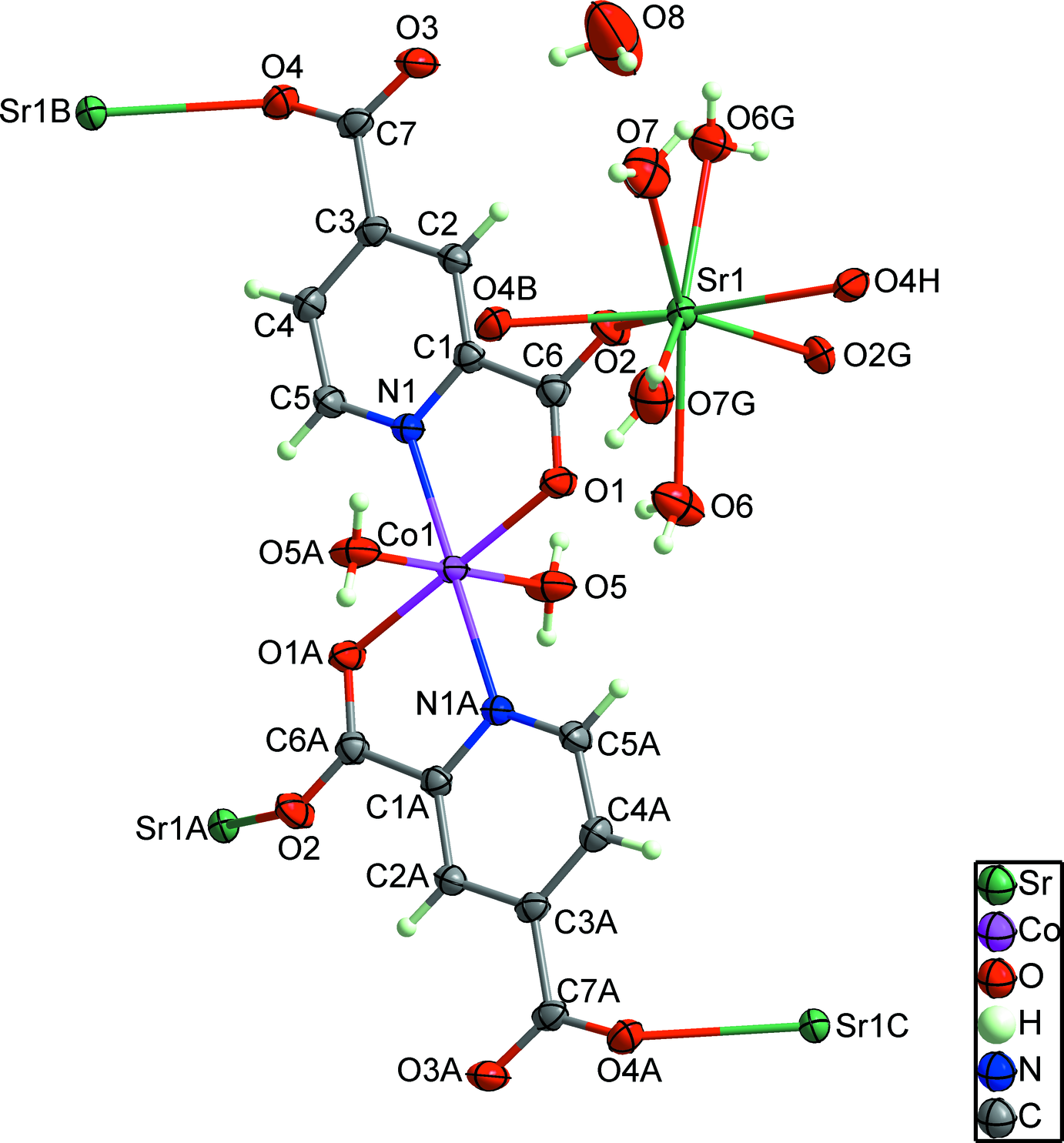

Supplement: Supplementary file 3 [file e-71-0m167-fig1.tif]

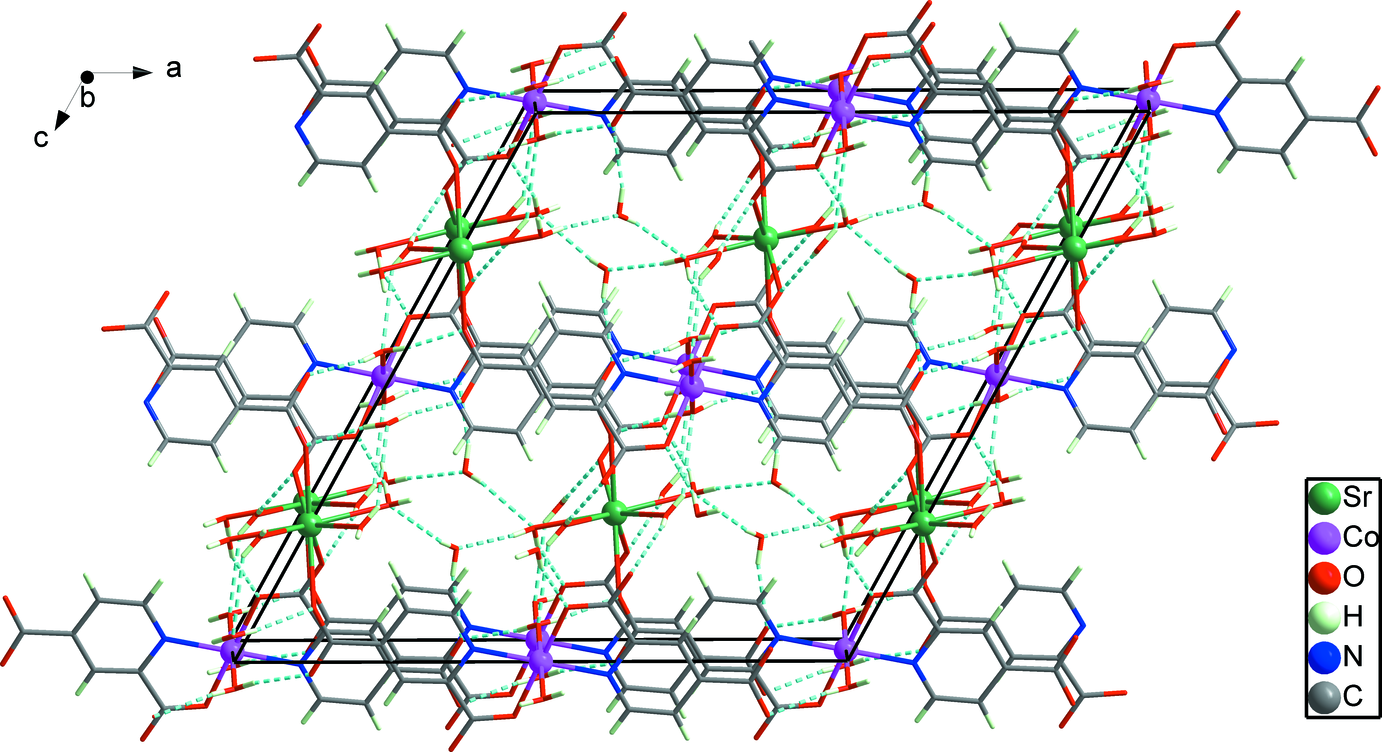

Supplement: Supplementary file 4 [file e-71-0m167-fig2.tif]
